# APTE: identification of indirect read-out A-DNA promoter elements in genomes

**DOI:** 10.1186/1471-2105-15-288

**Published:** 2014-08-26

**Authors:** David C Whitley, Valeria Runfola, Peter Cary, Liliya Nazlamova, Matt Guille, Garry Scarlett

**Affiliations:** Biophysics Laboratories, School of Biological Sciences, Institute of Biomedical and Biomolecular Science, University of Portsmouth, King Henry Building, King Henry I Street, Portsmouth, PO1 2DY UK; Centre for Molecular Design, School of Pharmacy and Biomedical Sciences, Institute of Biomedical and Biomolecular Science, University of Portsmouth, St Michael’s Building, White Swan Road, Portsmouth, PO1 2DT UK

**Keywords:** A-form DNA, Transcription, *Xenopus*, Promoter analysis

## Abstract

**Background:**

Transcriptional regulation is normally based on the recognition by a transcription factor of a defined base sequence in a process of direct read-out. However, the nucleic acid secondary and tertiary structure can also act as a recognition site for the transcription factor in a process known as indirect read-out, although this is much less understood. We have previously identified such a transcriptional control mechanism in early *Xenopus* development where the interaction of the transcription factor ilf3 and the *gata2* promoter requires the presence of both an unusual A-form DNA structure and a CCAAT sequence. Rapid identification of such promoters elsewhere in the *Xenopus* and other genomes would provide insight into a less studied area of gene regulation, although currently there are few tools to analyse genomes in such ways.

**Results:**

In this paper we report the implementation of a novel bioinformatics approach that has identified 86 such putative promoters in the *Xenopus* genome. We have shown that five of these sites are A-form in solution, bind to transcription factors and fully validated one of these newly identified promoters as interacting with the ilf3 containing complex CBTF. This interaction regulates the transcription of a previously uncharacterised downstream gene that is active in early development.

**Conclusions:**

A Perl program (APTE) has located a number of potential A-form DNA promotor elements in the *Xenopus* genome, five of these putative targets have been experimentally validated as A-form and as targets for specific DNA binding proteins; one has also been shown to interact with the A-form binding transcription factor ilf3. APTE is available from http://www.port.ac.uk/research/cmd/software/ under the terms of the GNU General Public License.

## Background

Transcription is the major level at which cellular protein concentration is regulated in response to environmental and developmental cues. Transcriptional control is mediated by the interaction of transcription factors and DNA elements. These elements are normally one instance of a set of similar sequences (or motifs) that the transcription factor ‘reads’ in a process known as direct read-out. There are some cases, however, where the transcription factor recognises not the sequence *per se* but the structure that the DNA adopts as a consequence of both sequence and conditions. The disruption of the DNA from the standard B-form conformation acts as a recognition site for the transcription factor in a process known as indirect read-out. This is well established in prokaryotes [1–3] but less recognised in eukaryotic cells, although an indirect read-out mechanism has been suggested for a selection of eukaryotic gene promoters [4–6]. Given the size of vertebrate genomes it is highly likely that some regions consist of sequences forming non-canonical structures and that some of these are regulatory. Indeed local DNA topography has been shown to correlate better than sequence with functional non-coding regions of the human genome [7].

The canonical double-stranded DNA structure is B-form, a right-handed helix with 3.4 Å between base pairs and a base tilt of 6 degrees to the helix axis. However, DNA can exist in a number of other conformations, the major types being A-form, Z-form and tetraplex, all of which have been implicated in gene regulation [8–10]. A-form is the canonical dsRNA structure with right-handed helices but with only 2.6 Å between bases and a 20-degree base tilt, while the sugar in A-form is in the c-3′ endo position in contrast to the c-2′ endo position observed for B-form. These differences lead to A-form helices being ‘shorter and fatter’, possessing major and minor grooves of similar width and the major groove deepened with respect to the B-form structure. Although DNA is usually in the canonical B-form it can be induced into A-form by dehydration and certain DNA sequences can naturally adopt an A-form helix under physiological conditions [11]. These A-form elements can then be specifically recognised by DNA binding proteins.

The interaction of the *Xenopus* CCAAT box transcription factor (CBTF) complex and the promoter of the developmentally important *gata2* gene is an example of a transcriptional regulatory mechanism involving A-form DNA. We have previously shown that this mechanism is based on an interaction requiring both DNA base specific (direct read-out) and DNA structure specific (indirect read-out) interactions [8, 6]. The CBTF complex is composed of approximately eight sub-units of which the ilf3 protein is currently the only published component; however, this subunit is critical for CBTF activity. Ilf3 is found in the nucleus when the *gata2* gene, a developmentally regulated gene involved in blood formation, is transcribed. A number of biochemical experiments have also confirmed ilf3 as a regulator of *gata2* transcription, including chromatin associated precipitation (ChIP) identifying ilf3 at the *gata2* promoter during active transcription of this gene [12]. Therefore the CBTF complex and its interactions is of interest both from developmental and transcriptionally mechanistic viewpoints.

Ilf3 contains two double stranded RNA binding domains (dsRBDs) and these domains are required for transcriptional activation *in vivo* and DNA binding *in vitro* [8]. The RNA binding activity of ilf3, and other dsRBD containing proteins, has been well characterised, indeed ilf3 was first identified through its interaction with RNA [13]. Crystal and NMR structures of a dsRBD alone exist [14], as does a crystal structure of the protein-RNA complex [15]. Alongside saturation mutagenesis studies, these structural studies have revealed that the domains recognise the A-form helical structure of double stranded RNA, although far less is known about their interaction with DNA. We have previously shown that *Xenopus* ilf3 contributes to the activity of CBTF as a transcriptional activator by its interaction with structure-specific DNA sequences. Specifically the dsRBDs of ilf3 are capable of interacting not only with A-form RNA but also non-canonical A-form DNA, such as that at the *gata2* promoter [6].

Here we report the development and validation of a bioinformatics tool for the analysis of genomic data to identify other potential promoters that utilise an A-form DNA structural component; in particular, those that are responsive to the transcription factor ilf3.

## Results and discussion

### Predicted promoter elements

**Figure 1:**
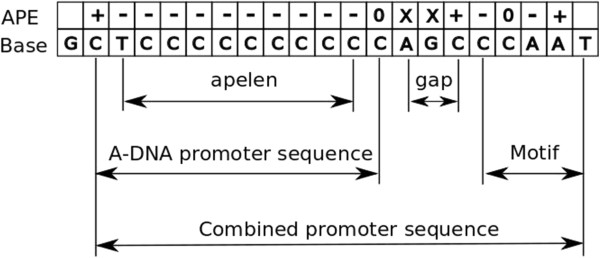
**A combined promoter sequence consists of an A-form promoter element followed by a direct read-out promoter motif.** The APE row indicates the signs of the APE values for the sequence in the Base row; with X denoting undetermined APE values [11]. The main parameters are the number of negative APE values in the APS (*apelen*), and the *gap* between the APS and the motif.

We implemented our search program based on the A-form prediction algorithm of Basham *et. al* [11] but including our previously described modifications [8]. This program was used to search the *Xenopus tropicalis* JGI 4.2 genome assembly for putative A-form promoters. Searches were further restricted to a 500 bp 5′ proximity of a start site of a transcribed unit and also to a variety of motifs known to be common transcription factor binding sequences. The prediction of A-form DNA is based on the *A-DNA propensity energy* (APE), a numerical measure derived from solvent free energy calculations that indicates the thermodynamic propensity for a sequence to adopt the A-DNA conformation. The APE value at position *i* in a DNA sequence depends on the central base *b*_*i*_ and the 5′ (*b*_*i-1*_) and 3′ (*b*_*i+1*_) flanking bases. From a triplet code of APE values for tri-nucleotides, the APE value for each base-pair is calculated (in kcal/mol) as the sum of the triplet APE values for the forward and reverse strands. In our process we have defined the predicted *A-form promoter sequence* (APS) as a sequence with consecutive negative APE values, together with the two flanking bases required for the APE calculation. Given a direct read-out promoter motif, for each gene the algorithm searches a region upstream of the transcription start site (TSS) for instances of the motif or its reverse complement preceded by an APS of pre-specified minimum length, with the APS and motif separated by at most a pre-specified maximum distance. The combined promoter sequence (CPS) extends from the start of the APS to the end of the motif (Figure 1).

We selected APS sequences of length ≥ 12 bp preceding several common promoter sequence motifs by at most 20 positions and within 500 bp of a TSS. A minimum APS of 12 bp was selected as our preliminary experimental studies show that this length of APS reliably gives an A-form structure as identified by circular dichroism (manuscript in preparation), while a limit of 20 bp between the APS and motif is based on the known footprint of the CBTF complex [8]. The number of APS and CPS (for the motifs CCAAT, GGGCGG, AGATA and TGATA) in the 4.2 assembly of the *Xenopus tropicalis* genome are shown in Table 1 along with their frequencies in regions 500 bp upstream of a TSS. The frequencies of the four motifs, in the whole genome and constrained to CPS or regions 500 bp upstream of a TSS, are shown in Table 2, the full list of hits is provided in Table 3. In general the CCAAT, AGATA and TGATA motifs occur with high frequency and in many cases several instances of a motif are found preceding a gene. The A-DNA promoter sequences, however, occur before only 3.2% of genes. An APS therefore occur only rarely in comparison with the motifs, and the combined promoter sequences only appear before approximately 0.47% of genes. Monte Carlo simulation of 10^6^ sequences of 500 bp selected randomly according to the nucleotide frequencies in the *Xenopus tropicalis* genome (0.299733 (A), 0.200318 (C), 0.200317 (G) and 0.299632(T)) produced expected numbers of 5.90 APS and 1.49 CPS in the genome. Thus we estimate that there are almost 100 times more APS and over 50 times more CPS in regions 500 bp upstream of TSS in the *Xenopus tropicalis* genome than would be expected by chance.

**Table 1:** **Frequency of A-DNA promoter sequences in Xenopus tropicalis 4.2 genome (apelen ≥ 10, motifgap ≤ 20, motifs for combined promoter sequences: CCAAT, GGGCGG, AGATA and TGATA)**

A-form promoter sequences (APS)	54,703
Combined promoter sequences (CPS)	9,909
Total number of genes in genome	18,442
Genes with APS within 500 bp upstream of TSS^a^	586 (3.18% of genes)
Genes with CPS within 500 bp upstream of TSS	86 (0.47% of genes)

^a^Transcription Start Site.

**Table 2:** **Frequency of motifs in combined promoter sequences (CPS) in** ***Xenopus tropicalis 4.2*** **genome (** ***apelen*** **≥ 10,** ***motifgap ≤ 20)***

Motif	CCAAT	GGGCGG	AGATA	TGATA
Genes with motif within 500 bp upstream of TSS^a^	13,255	2,531	12,703	12,201
Total number of motifs in genome	1,814,253	108,168	1,918,291	1,617,806
Motifs within 500 bp upstream of TSS (including multiples)	25,253 (1.39%)	3,377 (3.12%)	23,471 (1.22%)	20,927 (1.29%)
Motifs in CPS	3,771 (0.21%)	1,080 (1.00%)	2,351 (0.12%)	2,707 (0.17%)
Motifs in CPS within 500 bp upstream of TSS	36 (0.002%)	13 (0.012%)	18 (0.001%)	19 (0.001%)

^a^Transcription Start Site.

**Table 3:** **Gene IDs and names of the immediately downstream genes of the 86 putative A-form promoter elements identified in the JGI 4.2 genome assembly, the associated promoter motif sequence for each hit is shown alongside**

Gene ID	Gene name	Motif
ENSXETG00000003537	plcxd3	GGGCGG
ENSXETG00000008410	c5orf4	GGGCGG
**ENSXETG00000030719**	**unknown1**	**GGGCGG**
ENSXETG00000006282	unknown2	GGGCGG
ENSXETG00000003943	lrsam1	CCGCCC
ENSXETG00000027883	c3orf10	CCAAT
ENSXETG00000028111	unknown3	CCAAT
ENSXETG00000016171	gata2	CCGCCC
ENSXETG00000029861	unknown4	CCAAT
**ENSXETG00000009337**	**gdi3**	**CCAAT**
**ENSXETG00000012462**	**gtf2e1.2**	**CCAAT**
ENSXETG00000017744	XB-GENE-5853280	CCAAT
ENSXETG00000004674	eef1d	CCAAT
ENSXETG00000004472	mcts1	CCAAT
ENSXETG00000032447	LOC100488751	CCAAT
ENSXETG00000000668	xkr5	CCGCCC
ENSXETG00000033055	unknown5	CCAAT
**ENSXETG00000007609**	**thrsp**	**CCAAT**
ENSXETG00000002252	unknown6	CCAAT
ENSXETG00000026459	ywhaz	TATCA
ENSXETG00000029162	unknown7	TATCA
ENSXETG00000015053	gdpd5	TATCA
ENSXETG00000009868	tars	TATCA
ENSXETG00000010686	sepn1	TATCA
ENSXETG00000016524	LOC100493317	TATCT
ENSXETG00000018194	fam176a	TATCT
ENSXETG00000009404	adipor2	CCAAT
ENSXETG00000018026	sec22a	AGATA
**ENSXETG00000002371**	**kif27**	**AGATA**
ENSXETG00000010991	ercc4	TATCT
ENSXETG00000025304	unknown8	ATTGG
ENSXETG00000002603	gas2	TATCT
ENSXETG00000023254	zfp36l2.2	TATCA
ENSXETG00000009124	clcn7	CCAAT
ENSXETG00000018965	crat.1	CCAAT
ENSXETG00000027013	NP_001016033.1	CCAAT
ENSXETG00000027419	a4galt	TATCA
ENSXETG00000020165	mkrn2	CCAAT
ENSXETG00000029144	unknown9	ATTGG
ENSXETG00000030437	tnrc6a	ATTGG
ENSXETG00000018553	XB-GENE-5960869	TATCA
ENSXETG00000016062	znf184	GGGCGG
ENSXETG00000016933	ehmt1	ATTGG
ENSXETG00000014657	slc25a30	AGATA
ENSXETG00000003950	traf2	CCGCCC
ENSXETG00000030164	NP_001120021.1	AGATA
ENSXETG00000030426	unknown10	TATCA
ENSXETG00000022553	fam120a	ATTGG
ENSXETG00000007987	arg2	AGATA
ENSXETG00000023393	osbpl11	TGATA
ENSXETG00000017669	usp21	AGATA
ENSXETG00000013130	magi1	TATCT
ENSXETG00000023739	wrb	CCAAT
ENSXETG00000007387	bmi1	AGATA
ENSXETG00000016524	LOC100493317	ATTGG
ENSXETG00000013350	tfg	ATTGG
ENSXETG00000009412	unknown11	TATCT
ENSXETG00000000267	ccndx	CCAAT
ENSXETG00000010533	piwil2	ATTGG
ENSXETG00000007609	thrsp	TGATA
ENSXETG00000027421	HIST1H4G	TGATA
ENSXETG00000014657	slc25a30	ATTGG
ENSXETG00000014963	ctdsp1	TGATA
ENSXETG00000019650	myh11	AGATA
ENSXETG00000018194	fam176a	TATCT
ENSXETG00000029977	LOC100495404	ATTGG
ENSXETG00000008526	LOC100495179	GGGCGG
ENSXETG00000033908	UBE2U	AGATA
ENSXETG00000032885	P5F13_XENTR	ATTGG
ENSXETG00000019263	pdss2	CCAAT
ENSXETG00000008969	rad51l3	TATCA
ENSXETG00000022325	unknown12	TATCA
ENSXETG00000020057	utp6	CCAAT
ENSXETG00000007609	thrsp	TATCT
ENSXETG00000013463	zmynd12	ATTGG
ENSXETG00000015404	shc1	TATCT
ENSXETG00000027433	otop2	ATTGG
ENSXETG00000021081	sgcg	GGGCGG
ENSXETG00000006922	ss18	TATCA
ENSXETG00000033607	asxl1	CCAAT
ENSXETG00000023477	hdhd3	ATTGG
ENSXETG00000003248	strada	TGATA
ENSXETG00000033920	F166B_XENTR	CCGCCC
ENSXETG00000010684	dnajc19	TGATA
ENSXETG00000027998	prss8	CCGCCC
ENSXETG00000010250	chrnb3	TGATA

Those selected for analysis are marked in bold.

### Selection and validation of a predicted promoter

**Figure 2:**
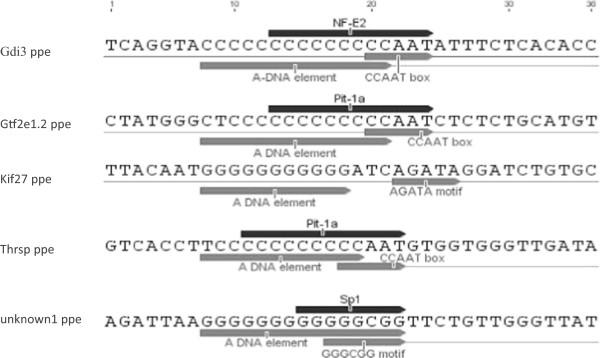
**The five selected sequences and their predicted binding proteins.** Each of the putative promoter elements (ppe) sequences are within 500 bp 5′ of the transcription start site of the genes-gdi3, gtf2, kif27, thrsp and unknown1, The key elements with potential gene regulatory function are underlined with grey arrows. The black arrow above each oligonucleotide indicates a putative transcription factor binding site and its direction of binding. The putative transcription factor binding sites were predicted using the EMBOSS database run through Geneious R7 7.1.4.

Having identified 86 putative promoter elements in the JGI 4.2 assembly we randomly selected five for validation. The 36 bp sequences corresponding to the five selected CPSs are shown in Figure 2 with their predicted transcription factor binding sites. Experimentally we confirmed that these sequences were (i) A-form in character and (ii) indeed a target for a DNA binding protein.

Circular Dichroism experimental studies of all five selected sequences confirm that these GC-rich duplexes are largely in the A-form conformation. The data shows two strong positive bands with maxima between 186-189 nm and 267-269 nm respectively for all five constructs with a negative band minima between 240-243 nm, these spectra are indicative of A-form. The absence of a clear, strong positive band at 180-186 nm suggest there is little B-form DNA duplex present in any of the five sequences, although there is weak positive contribution between 180-190 nm for thrsp, obp, kif27 and gdi3 causing a slight distortion to the main positive band (260 nm to 300 nm). Further, the intensity of the band maxima at (267-269 nm) is significantly more positive than expected for B-form (+2.5 to 3.3) and the experimental ellipticity values are more typical of A-form duplexes (+4.3 to 6.86). Using the triple base APE prediction for A and B-form DNA duplexes all five selected DNA sequences have strong continuous A-form runs upstream of the CCAAT, AGATA and GGGCGG motifs. These continuous A-form regions only represent 28 to 39% of the total duplexes in the A-form for all five sequences, the CD measurements suggest that the A-form content is at least between 50 to 80% for all five duplexes. Using the triple base APE prediction for A and B-form Dna duplexes the total A-form prediction content for Gtf2e1.2 for example is 56% with 20% having no bias for A or B-form, 14% undetermined APE values, 11% with a preference for B-form duplexes. This would suggest the minimum A-form content is 56% and may be as high as 85%, however in all cases the duplexes are mainly in the A-form conformation.

**Figure 3:**
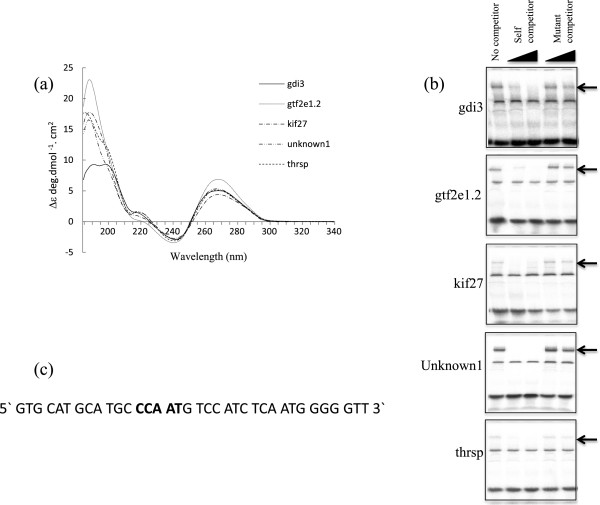
**The putative promoter element is A-form and binds ilf3** ***in vitro*** **. (a)** Duplex 36 bp oligonucleotides corresponding to the five identified putative promoter elements display A-form DNA characteristics as observed by circular dichroism. **(b)** These duplex oligonucletides are shifted in EMSA experiments, these complexes are competed by titration of unlabelled self-competitor but not by CCAAT box containing B-form duplexes. The specific complexes are indicated by arrows. **(c)** The sequence of the B-form competitor used in the EMSA is shown, the CCAAT box is indicated in bold.

We next tested that these oligonucleotides were specific targets for DNA binding proteins such as transcription factors. Radiolabelled sequences were mixed with whole embryo extract and electrophoretic mobility shift (EMSA) assays were performed. All the sequences found specific complexes with embryo extract, these complexes were competed by unlabelled self-competitor. However they were not competed by an alternative 36 bp competitor that contained a CCAAT box sequence but which was strongly B-form in structure (Figure 3a and b). Having shown that all five of the selected sequences were both A-form and targets for specific DNA binding proteins we selected the gdi3 putative promoter, which contains a direct (i.e. present on the same strand as the downstream gene coding strand) CCAAT motif, for further characterisation and to test if it was also a target of the ilf3 containing transcription factor complex CBTF.

**Figure 4:**
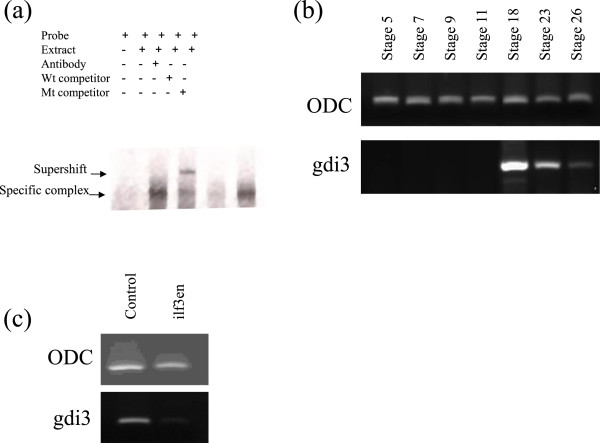
**The expression of** ***gdi3*** **mRNA is maximal at neurula stage and can be modulated by ilf3. (a)** The complex gdi3 specific complex can be supershifted by addition of anti-ilf3 antibody. **(b)** The *gdi3* gene expression is zygotic and peaks at neurula stage 18 when ilf3 is known to be nuclear and active. **(c)** Expression of *gdi3* is ablated relative to an internal control, ODC, by exogenous expression a dominant-negative form of ilf3 (ilf3en) which acts at the transcriptional level.

Upon co-incubation of an antibody raised against ilf3 the gdi3 complex was supershifted in EMSA, confirming the presence of ilf3 in the nucleic acid-protein complex (Figure 4a). The role of the gdi3 putative promoter element was also tested *in vivo*. To this end the expression profile of *gdi3* was analysed using RT-PCR. Expression of *gdi3* mRNA is absent until stage 11, then is expressed between stage 12 and 18, the latter of which it is at maximal, and from which its expression levels decrease until the last point sampled at stage 26 (Figure 4b). This expression wave occurs just after the maximal expression of *gata2*, a gene that is also controlled by the ilf3 transcription factor. A dominant-negative form of ilf3 (ilf3en) uses the fusion of ilf3 to the engrailed domain from *Drosophila* to repress transcription from any ilf3 binding site by recruitment of histone deacetylases [16]. This fusion has been shown to down-regulate *gata2* mRNA levels when exogenouly expressed in *Xenopus tropocalis* embryos [8]. Synthetic mRNA encoding ilf3en was micro-injected into one-cell stage embryos before harvesting at stage 18 and total RNA was extracted, RT-PCR was again used to analyse levels of *gdi3* mRNA. Expression of *gdi3* was ablated relative to levels of engrailed alone injected controls (Figure 4c), indicating ilf3 is involved in regulation of *gdi3 in vivo* at a transcriptional level.

## Conclusion

We have previously identified and characterised a promoter element that requires an unusual A-form DNA structure in conjunction with a known promoter sequence motif. This combination of direct and indirect read-out mechanism drives early embryonic expression of the *gata2* gene in *Xenopus* and is responsive to the ilf3 containing transcription factor complex CBTF. However, the question of the prevalence of this type of regulatory mechanism in genomes remained. To address this we implemented a Perl program to investigate the occurence and used this to search the 4.2 version of the *Xenopus* genome. From the 86 hits obtained we selected five to test for both actual A-form structure and as specific targets for DNA binding proteins. All five of the selected targets were experimentally validated as A-form and as protein binding sites. One of these five, containing a CCAAT motif as does the previously identified *gata2* promoter, was selected for further validation. This element is the putative promoter for the *gdi3* gene and was shown by supershift to be a target for the known *gata2* transcription factor ilf3. The temporal expression pattern of *gdi3* occurs shortly after that of *gata2* and gdi3 transcription is also responsive to ilf3 fusion proteins *in vivo*. Taken together this is strong evidence for the element identified by the program to be a critical component of the promoter of *gdi3*.

Identification of the promoter elements required the A-forming potential of a base triplet of a given sequence to be calculated in a moving window along the genome using the method of Basham *et. al.* In the overwhelming majority of hits the APS consists of a consecutive sequence of Cs or Gs, with the first or second position in a block of Cs occasionally replaced by a T. Only five cases were observed where this pattern does not hold, all involving repeated blocks of ATGC. However, it should be noted that APE values do not exist for 14 of the 64 possible triplets, which are effectively ignored by the present algorithm. The reliability of the method would no doubt be increased if these non-determined values were assigned. Despite this, ***apte*** provides a powerful tool for potential identification of A-form regulatory elements in whole genomes. A major problem in eukaryotic transcriptional studies is that transcription factor binding sites occur with high frequency and this leads to many ‘false positive’ identification of promoter elements by search programs. Potentially by considering DNA structure the reliability of such search programs could be significantly enhanced. For instance there are 25,253 CCAAT sequences (counting multiples per gene) within 500 bp of a TSS in the 4.2 genome and 54,703 APS sequences anywhere in the genome. However there are only 36 in conjunction, a far more manageable number to screen.

Previous work on indirect read out mechanisms invoved with DNA recognition has largely been limited to *in vitro* experiments. Our validation of *gdi3* as being regulated by such a mechanism is at least partially *in vivo*. Within eukaryotic genomes DNA is chromatinised with the interactions of the histones and the DNA, providing not only packaging but regulatory functions. It is unclear how non B-form DNA structures affects chromatinisation, possibly they chromatinise less well and are therefore bare regions at promoters, but the fact that we have identified a gene that is regulated *in vivo* by an A-form binding protein suggests that these structures persist within the chromatin environmment.

Although our results reflect mainly the identification of genes responsive to the ilf3 transcription factor potentially other A-form DNA binding proteins may also be recognising these elements. Importantly, the ability to look at whole genome assemblies means that it is now possible to study the role of these A-form elements within gene regulatory networks.

## Methods

### Algorithm and implementation

The algorithm is implemented as a Perl program named **apte** (A-form promoter transcription elements), which provides both a command-line interface and a Perl/Tk graphical interface. The program reads genomic sequence data from General Feature Format (GFF) Version 3 files (http://www.sequenceontology.org/gff3.shtml) and from Ensembl MySQL databases (http://www.ensembl.org/info/data/ftp/index.html). GFF input files should contain a list of genes to be searched and the DNA sequence in FASTA format. Access to Ensembl databases is provided through the Ensembl Perl API (http://www.ensembl.org/info/docs/api/index.html) which is a prerequisite for the program.

The main input parameters for ***apte*** are: *motif*, the promoter motif sequence; *apelen*, the minimum number of negative APE values in the APS; *motifgap*, the maximum number of bases between the APS and the motif; and *genegap*, the size of the region preceding the TSS to be searched. The default values adopted for the parameters are *motif* = CCAAT, *apelen* = 10, *motifgap* = 20 and *genegap* = 500. Searches can cover an entire genome or be limited to a specific gene or sequence region. Searches can also be made solely for A-DNA promoter sequences or promoter motifs. Results are output as a tab-separated table with a row for each combined sequence found, listing the APS and motif positions and summary details of the corresponding gene. Options are provided to write the results in GFF format; or in BED or WIG format files which may be uploaded to the Ensembl genome browser for display as custom tracks. The BED files indicate the location of the APS, the motif and the sign of the APE values over the search region. The WIG files plot the APE scores over the search region.

### Microinjection and RT-PCR

*Xenopus* embryos were collected at time points during early developmental stages according to Nieuwkoop [17] and RNA extracted for RT-PCR analysis using the method of Steinbach and Rupp [18]. The samples were amplified to the linear phase of the amplification with the ODC gene used as an internal control, all primer sequences are available in supplemental information. Synthetic mRNA was prepared as previously described [8] and injected into both cells of two-cell stage embryos.

### Circular dichroism

An Applied Photophysics Pi* 180 instrument was flushed with nitrogen gas (Oxygen-Free) for all CD experiments. Cell pathlengths of 1 mm and 4 mm were used to obtain far and near ultra-violet data respectively. Each duplex was dissolved in 100 mM KF 5 mM NaPO4 buffer pH 7.6 at room temperature and stored on ice. Concentrations were determined by UV measurements at 260 nm coupled with snake-venom phosphodiesterase time course digestions to correct for hypochromic difference. The samples were run at 20+/-0.1C using a Melcor Peltier Thermoelectric Temperature Control Unit. Data was collected every 1 nm over the wavelength range 183 nm to 360 nm using adaptive sampling in conjunction with signal averaging in all cases. The instrument wavelength accuracy was 0.1+/-nm determined from the Xeon lines and the ellipticity was calibrated from camphor suphonic acid at 290.5 nm.

### Electrophoretic mobility shift assay (EMSA)

DNA oligonucleotides (Invitrogen) were annealed to form duplexes and end-labeled by T4 polynucleotide kinase (NEB) using γ^33^P ATP. The proteins were incubated with the nucleic acid probe for 15 minutes on ice in EMSA buffer [19] in the presence of 500 ng poly dI-dC. Either wild-type or mutant non-labeled competitor was added at a 50 times excess to two of the reactions while a third reaction was incubated with anti-ilf3 antibody to allow identification of the specific DNA-protein complex. After incubation the DNA and DNA-protein complexes were separated on a 4% native polyacrylamide gel in 0.25 X TBE. The gels were dried and visualized using a phosphorimager (Fuji).
